# The neurodevelopmental disorder risk gene *DYRK1A* is required for ciliogenesis and control of brain size in *Xenopus* embryos

**DOI:** 10.1242/dev.189290

**Published:** 2020-06-22

**Authors:** Helen Rankin Willsey, Yuxiao Xu, Amanda Everitt, Jeanselle Dea, Cameron R. T. Exner, A. Jeremy Willsey, Matthew W. State, Richard M. Harland

**Affiliations:** 1 Department of Psychiatry and Behavioral Sciences, Langley Porter Psychiatric Institute, Quantitative Biosciences Institute, and Weill Institute for Neurosciences University of California San Francisco, San Francisco, CA 94143, USA; 2 Department of Molecular and Cell Biology, University of California, Berkeley, CA 94720, USA; 3 Department of Psychiatry and Behavioral Sciences, Institute for Neurodegenerative Diseases, Quantitative Biosciences Institute, and Weill Institute for Neurosciences, University of California San Francisco, San Francisco, CA 94143, USA

**Keywords:** DYRK1A, Ciliogenesis, Autism, Down Syndrome, Microtubules, Spindle

## Abstract

*DYRK1A* [dual specificity tyrosine-(Y)-phosphorylation-regulated kinase 1 A] is a high-confidence autism risk gene that encodes a conserved kinase. In addition to autism, individuals with putative loss-of-function variants in *DYRK1A* exhibit microcephaly, intellectual disability, developmental delay and/or congenital anomalies of the kidney and urinary tract. *DYRK1A* is also located within the critical region for Down syndrome; therefore, understanding the role of *DYRK1A* in brain development is crucial for understanding the pathobiology of multiple developmental disorders. To characterize the function of this gene, we used the diploid frog *Xenopus tropicalis*. We discover that Dyrk1a is expressed in ciliated tissues, localizes to ciliary axonemes and basal bodies, and is required for ciliogenesis. We also demonstrate that Dyrk1a localizes to mitotic spindles and that its inhibition leads to decreased forebrain size, abnormal cell cycle progression and cell death during brain development. These findings provide hypotheses about potential mechanisms of pathobiology and underscore the utility of *X. tropicalis* as a model system for understanding neurodevelopmental disorders.

## Teaser

**Summary:** The autism and Down syndrome risk gene *DYRK1A* localizes to ciliary components and mitotic spindles, and is required for ciliogenesis and cell cycle control during embryonic *Xenopus* development.

## INTRODUCTION

*DYRK1A* [dual specificity tyrosine-(Y)-phosphorylation-regulated kinase 1 A] encodes a highly conserved serine-threonine protein kinase expressed during human embryonic brain development (van Bon et al., 2016). Large-scale human genetics efforts have rigorously associated *DYRK1A* mutations with numerous developmental disorders (van Bon et al., 2015; Feki and Hibaoui, 2018). Specifically, *DYRK1A* is a high-confidence risk gene for autism spectrum disorders (ASDs) (Satterstrom et al., 2020; Iossifov et al., 2012). More recently, *DYRK1A* haploinsufficiency has been proposed to cause a distinct syndrome, characterized by ASD along with microcephaly, intellectual disability, developmental delay, and/or congenital anomalies of the kidney and urinary tract (van Bon et al., 2016; Earl et al., 2017; Ji et al., 2015; Blackburn et al., 2019). *DYRK1A* is located within the Down syndrome critical region on chromosome 21, and increased doses of *DYRK1A* have been implicated in Down syndrome pathobiology (Arron et al., 2006). Accordingly, DYRK1A kinase inhibitors are currently being explored for their therapeutic potential (Neumann et al., 2018; Feki and Hibaoui, 2018; Duchon and Herault, 2016).

The function of *DYRK1A* in brain development has been studied in a wide range of model systems. The microcephaly observed in humans with *DYRK1A* haploinsufficiency is recapitulated in both *Drosophila* and mouse heterozygous mutant animals (Fotaki et al., 2002; Tejedor et al., 1995). Mice with *Dyrk1a* heterozygous mutations also display motor deficits, developmental delay and altered behavior (Fotaki et al., 2002; Dierssen and de Lagrán, 2006; Arqué et al., 2009). At the cellular level, there is evidence that *DYRK1A* is required for cell cycle control, differentiation and dendritic spine development (Shaikh et al., 2016; Ori-McKenney et al., 2016; Dang et al., 2018). Molecularly, DYRK1A has been shown to phosphorylate a wide range of target proteins, including mRNA splicing factors (de Graaf et al., 2006; Shi et al., 2008), transcription factors (Fernandez-Martinez et al., 2009; Ehe et al., 2017; Mao et al., 2002), cyclins (Smith and Calegari, 2015; Soppa et al., 2014; Najas et al., 2015; Chen et al., 2013), and β-tubulin (Ori-McKenney et al., 2016). However, it is unknown which of these functions are central to the role of DYRK1A in brain development and which underlie pathobiology of related conditions.

Here we characterize the expression, localization and function of Dyrk1a in *Xenopus tropicalis* embryos to better understand its role in development. We uncover a novel localization of Dyrk1a to ciliary components and a corresponding novel requirement for Dyrk1a in ciliogenesis. Furthermore, we describe a novel localization of Dyrk1a to mitotic spindles, and demonstrate that inhibition alters cell cycle progression and cell survival in the developing brain. These observations suggest underlying deficits in microtubule dynamics, for which Dyrk1a has a known role (Ori-McKenney et al., 2016). Finally, we observe a marked reduction in forebrain size following *dyrk1a* loss of function, a phenotype consistent with human *DYRK1A* haploinsufficiency and deficits in cell cycle progression.

## RESULTS AND DISCUSSION

### Expression and localization of Dyrk1a during *X. tropicalis* development

**Fig. 1.**
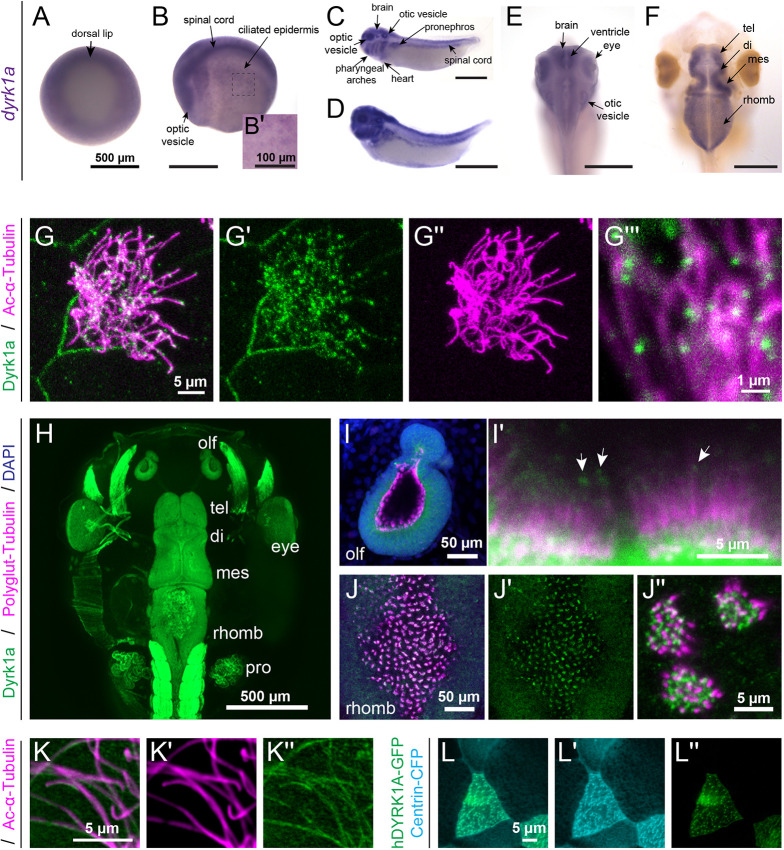
**Expression and localization of Dyrk1a during *X. tropicalis* development.** (A-F) RNA *in situ* hybridization in *X. tropicalis*. (A-B′) *dyrk1a* is expressed during gastrulation (stage 10.5, A) and at stage 20 (B) in the brain, spinal cord, optic vesicles and ciliated epidermis (B′). (C,D) At stages 32 (C) and 35 (D), *dyrk1a* is expressed in the brain, spinal cord, otic vesicles, optic vesicles, pharyngeal arches, heart, epidermis and pronephros. (E,F) At stages 40 (E) and 46 (F), *dyrk1a* is expressed in the brain, especially along ventricles [telencephalon (tel), diencephalon (di), mesencephalon (mes) and rhombencephalon (rhomb)]. Scale bars: 500 µm; 100 µm in B′. (G-G‴) Embryonic epidermis antibody staining for Dyrk1a protein (green) shows puncta along ciliary axonemes labeled for acetylated α-Tubulin (Ac-α-Tubulin, magenta). See Fig. S1 for antibody validation. (H) Dorsal view of whole-mount antibody staining showing Dyrk1a (green) throughout the nervous system and in the pronephros (pro). (I-J″) Dyrk1a (green) is on cilia marked by polyglutamylated-Tubulin (polyglut-Tubulin, magenta) in the olfactory epithelium (olf, I,I′) and in the rhombencephalon (J-J″). White arrows (I′) indicate the puncta of Dyrk1a (green) on cilia. There is also strong membrane staining. (K-K″) Human GFP-tagged DYRK1A (hDYRK1A-GFP, green) localizes in puncta along ciliary axonemes labeled for acetylated α-Tubulin (magenta). (L-L″) hDYRK1A-GFP (green) colocalizes with Centrin-CFP (cyan) at basal bodies.

By whole-mount RNA *in situ* hybridization, we detected *dyrk1a* mRNA throughout *X. tropicalis* embryonic development, spanning gastrulation (Fig. 1A), neurulation (Fig. 1B) and organogenesis (Fig. 1C-F). Expression is strong in neural and ciliated tissues, including the embryonic epidermis (Fig. 1B), the developing brain and eye, the otic vesicle, and the pronephros (Fig. 1C-E; Blackburn et al., 2019). It is also expressed in the pharyngeal arches and developing heart (Fig. 1C,D). During neurogenesis, it is highly expressed in the proliferative cells lining the brain ventricles (Fig. 1E). Later, it is expressed throughout the tadpole brain, in the telencephalon (tel), diencephalon (di), mesencephalon (mes) and rhombencephalon (rhomb) (Fig. 1F). 

By antibody staining of tailbud stages, we detected endogenous *Xenopus* Dyrk1a protein in puncta along ciliary axonemes, labeled by acetylated α-Tubulin (Fig. 1G). This staining is lost following *dyrk1a* depletion by morpholino oligonucleotides (MO) or by CRISPR/Cas9-mediated mutagenesis (Fig. S1). In tadpole stages, Dyrk1a antibody staining shows specific labeling throughout the brain (Fig. 1H), and especially in ciliated cells such as the olfactory epithelium (olf, Fig. 1I) and the roof of the fourth ventricle in the rhombencephalon (rhomb, Fig. 1J). We confirmed ciliary localization with a GFP-tagged human DYRK1A (hDYRK1A-GFP) expressed in the *Xenopus* embryonic epidermis. Human DYRK1A also localized in puncta along ciliary axonemes (Fig. 1K) and at ciliary basal bodies labeled with Centrin-CFP (Fig. 1L). These results demonstrate that Dyrk1a is expressed in neural and ciliated tissues during embryonic development and localizes to ciliary axonemes.

### *dyrk1a* is required for ciliogenesis and telencephalon size

**Fig. 2.**
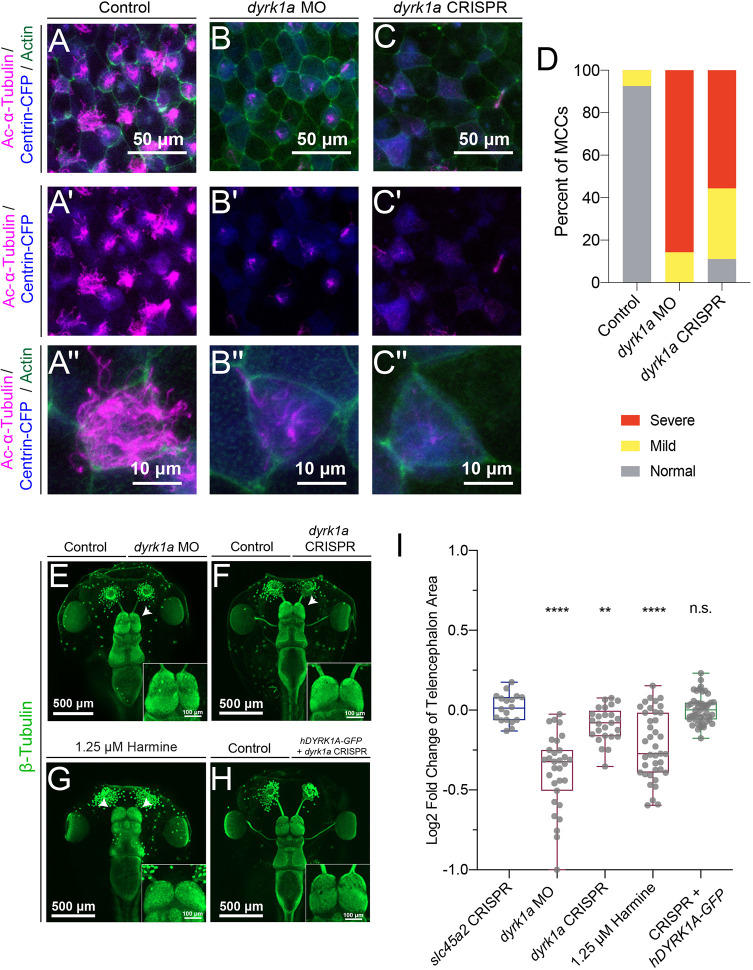
***dyrk1a* is required for ciliogenesis and brain size.** (A-C″) Stage 35 *X. tropicalis* embryonic epidermis stained for acetylated α-Tubulin (cilia, magenta), phalloidin (actin, green) and expressing Centrin-CFP (basal bodies, blue). Injection of *dyrk1a* morpholino (MO) (B-B″) or CRISPR/Cas9 reagents (C-C″) causes a loss in cilia (magenta) compared with the injected control (A-A″). See also Figs S2, S3. (D) Quantification of ciliogenesis phenotype (severe, mild and normal) in multiciliated cells (MCCs) by condition. *n*>35 for each condition. (E-H) Dorsal view of β-Tubulin antibody staining (green) of *X. tropicalis* tadpoles, injected (right side) with *dyrk1a* morpholino (MO) (E) or *dyrk1a* CRISPR (F), or co-injected with *dyrk1a* CRISPR and human GFP-tagged DYRK1A (hDYRK1A-GFP) (H). (G) Dyrk1a kinase inhibitor harmine-treated embryos are affected bilaterally (1.25 µM). Telencephalon regions are shown in insets. White arrowheads indicate telencephalon size phenotypes. (I) Quantification of log2 fold change of telencephalon size normalized to control. Whiskers indicate maximum and minimum values, boxes show the interquartile range and the line is the median. Every point is the value for one animal. *P*-values were calculated using non-parametric Mann–Whitney rank sum tests in comparison with the negative control CRISPR targeting pigmentation gene *slc45a2*. *P*<0.0001 for harmine in comparison with paired DMSO treatment. **P<0.01; ****P<0.0001; n.s., not significant (*P*>0.05).

To investigate the function of Dyrk1a, we disrupted endogenous expression with either MO knockdown or CRISPR/Cas9 mutagenesis. This *dyrk1a* translation-blocking MO has previously been validated in *Xenopus* kidney development (Blackburn et al., 2019). We confirmed that the single guide RNA (sgRNA) targeting *dyrk1a* was efficient by Sanger sequencing and sequence deconvolution (mean efficiency 71%, standard deviation 18%; Fig. S2). In both cases, these loss of function strategies abolished Dyrk1a antibody staining in the ciliated epidermis in tailbud stages (Fig. S1). Therefore, we assayed whether ciliogenesis was disrupted following loss of *dyrk1a* by either strategy. In both cases, we observed that depletion of Dyrk1a during embryonic development leads to ciliogenesis defects (Fig. 2A-D, Fig. S3). 

Because individuals with *DYRK1A* haploinsufficiency have microcephaly, we next assayed the gross brain anatomy of *dyrk1a* loss-of-function animals at the tadpole stages by labeling neurons with β-Tubulin antibody staining. We injected MO or CRISPR/Cas9 components into one cell at the two-cell stage, generating unilateral loss of *dyrk1a* function, where the uninjected half serves as a contralateral control (Willsey et al., 2018b; DeLay et al., 2018; Lasser et al., 2019). Loss of *dyrk1a* function by either method led to a reduction in telencephalon size without a dramatic alteration in gross regional anatomy (Fig. 2E-F,I, both *P*<0.05 by Mann–Whitney rank sum test compared with the negative control pigmentation gene *slc45a2*). In both this assay and in the ciliogenesis assay, the MO injection produced a stronger effect than the CRISPR injection, potentially because of the presence of maternal *dyrk1a* mRNA (Owens et al., 2016) that will only be targeted by the MO. We were able to rescue the reduction in telencephalon size following CRISPR injection by co-injection of *hDYRK1A-GFP* (Fig. 2H-I), suggesting conservation of function. Finally, we validated this loss-of-function phenotype by inhibition of Dyrk1a kinase activity using the validated pharmacological inhibitor harmine (Göckler et al., 2009) (Fig. 2G, *P*<0.05). Together, these results demonstrate that *dyrk1a* is required for ciliogenesis and regulation of telencephalon size control during *Xenopus* embryonic development.

### RNA-sequencing implicates cell cycle control in *dyrk1a* brain phenotype

**Fig. 3.**
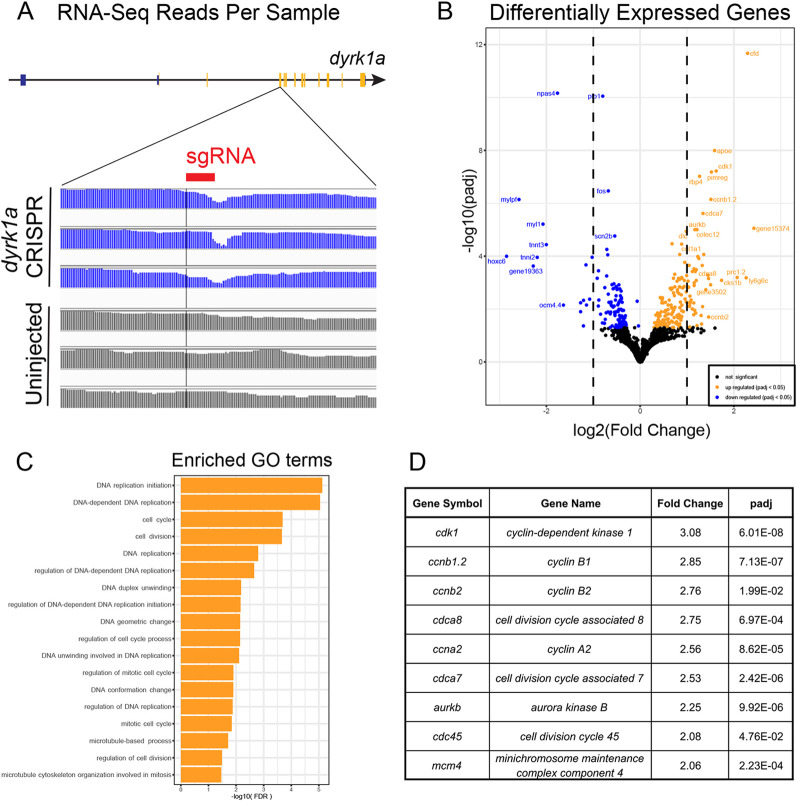
**RNA-sequencing implicates cell cycle control in the *dyrk1a* brain phenotype.** (A) RNA-sequencing reads of uninjected control and *dyrk1a* CRISPR samples. These were from dissected stage 46 brains. Sequencing reads (blue) are lost adjacent to the sgRNA target site (red) in the injected samples but not the uninjected samples within the targeted exon of *dyrk1a*. (B) Volcano plot of differentially expressed genes (DEGs) following *dyrk1a* mutation with fold change and adjusted *P*-value in log scales on the *x*- and *y*-axes. Upregulated (orange) and downregulated (blue) DEGs with *P*<0.05 are shown. (C) Enriched gene ontology (GO) terms of DEGs by false discovery rate (FDR). (D) A list of selected DEGs with high fold change and high significance related to cell cycle control.

To understand how *dyrk1a* loss of function leads to a smaller telencephalon, we sequenced RNA from dissected brains from control and *dyrk1a* CRISPR-injected animals at stage 46 (Fig. 3). After sequencing, we first confirmed that CRISPR/Cas9 mutagenesis resulted in mRNA disruption by analyzing sequence reads surrounding the protospacer adjacent motif (PAM) site within the target exon of *dyrk1a*. We observed a depletion of sequencing depth in all three injected replicates but not in control replicates (Fig. 3A), suggesting efficient targeting. Next, we identified 294 differentially expressed genes (Fig. 3B, Table S1), of which 172 were upregulated (orange points) and 122 were downregulated (blue points). Gene ontology enrichment analysis of the differentially expressed genes demonstrated strong enrichment in biological processes related to cell cycle control, DNA replication and microtubules (Fig. 3C, Table S2). Strikingly, canonical cell cycle genes such as cyclin-dependent kinase 1, aurora kinase B, cyclin B1, cyclin B2 and cyclin A2 are all increased greater than twofold (Fig. 3D, Table S1). Therefore, we hypothesized that the small telencephalon phenotype may be due to disruption of cell cycle progression. 

**Fig. 4.**
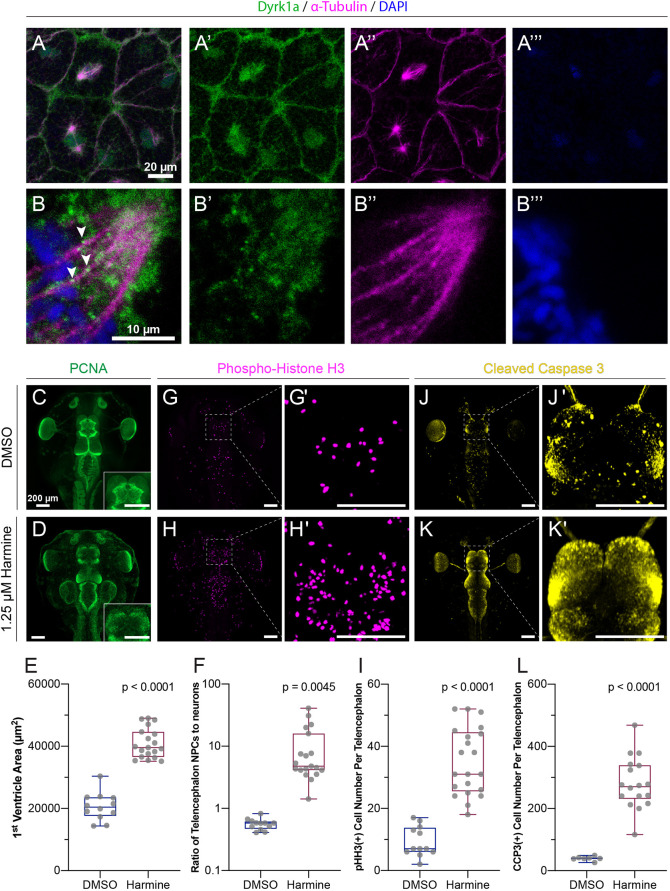
**Dyrk1a localizes to mitotic spindles and is required for cell cycle progression and survival.** (A-B‴) Stage 10 embryos stained for Dyrk1a (green), α-Tubulin (microtubules, magenta) and DAPI (DNA, blue). White arrowheads indicate Dyrk1a puncta near mitotic spindle. (C,D,G,H,G′,H′,J,J′,K,K′) Dorsal view of *X. tropicalis* tadpoles treated with DMSO (top row) or 1.25 µM Dyrk1a inhibitor harmine (bottom row). (C,D) Antibody staining for PCNA (proliferating cell nuclear antigen, S-phase marker, green). Telencephalon region is shown in the inset. (E) Quantification of first ventricle area using PCNA staining. (F) Quantification of the ratio of neural progenitor cells (NPCs, PCNA area) to the area of differentiated neurons in each telencephalon (log scale). (G,H) Antibody staining for pHH3 (phospho-histone H3, M-phase marker, magenta). (G′,H′) High-magnification views of telencephalon regions from G and H. (I) Quantification of pHH3-positive cell number per telencephalon. See also Fig. S4. (J,K) Antibody staining for CCP3 (cleaved caspase 3, cell death marker, yellow). (J′,K′) High-magnification view of telencephalon from J and K. (L) Quantification of CCP3-positive cell number per telencephalon. Scale bars: 200 µm. Whiskers are maximum and minimum values, boxes show the interquartile range and the line is the median. Every point is the value for one animal. *P*-values were calculated using non-parametric Mann–Whitney rank sum tests.

It has previously been shown that loss of *dyrk1a* speeds the transition from gap 1 (G1) phase to synthesis (S) phase due to its role in regulating Cyclin D degradation (Chen et al., 2013; Najas et al., 2015; Soppa et al., 2014). However, our observations also suggest a novel role for Dyrk1a in regulating mitotic (M) phase. Specifically, we observed upregulation of cyclin B1 and cyclin B2, two genes known to reach maximum mRNA expression during the M phase of the cell cycle (Ito, 2000). We also observed enrichment of microtubule-related processes, including gene ontology terms related to the mitotic spindle. These data, combined with the fact that Dyrk1a localizes to ciliary axonemes and is required for ciliogenesis, a process that requires dynamic microtubule remodeling, led us to hypothesize that Dyrk1a might impact M phase via a role in mitotic spindle dynamics. This idea is consistent with a previously described function for Dyrk1a in regulating microtubule dynamics in developing dendrites (Ori-McKenney et al., 2016). Therefore, we stained blastula stage embryos, which are highly mitotic, for endogenous Dyrk1a. Remarkably, we observed a clear and novel localization near mitotic spindles marked by α-Tubulin (Fig. 4A,B). Together, these findings suggest that *dyrk1a* is important for cell cycle progression, possibly by modulating microtubule dynamics and/or the mitotic spindle, and that loss may cause stalling during the M phase of the cell cycle. 

### Dyrk1a is required for cell cycle progression and survival

Next, we used the validated Dyrk1a kinase inhibitor harmine to determine whether cell cycle progression is indeed affected following loss of Dyrk1a function. This perturbation also results in the small telencephalon phenotype seen with the genetic inhibitors (Fig. 2G,I), but has the advantage of enhanced temporal control, and, thus, the ability to bypass early embryonic roles of Dyrk1a. Consistent with the RNA-seq data and the known role of Dyrk1a in regulating Cyclin D, we observed an increase in the number of cells expressing the S-phase marker PCNA (proliferating cell nuclear antigen) in the developing brain (Fig. 4C-E, *P*<0.0001 by Mann–Whitney rank sum test) as well as a relative increase in these cells compared with the rest of the telencephalon (Fig. 4F, *P*=0.0045). Dyrk1a inhibition also caused an increase in cells labeled by phospho-histone H3 (pHH3), a marker for M phase (Fig. 4G-I, *P*<0.0001, Fig. S4C,D), suggesting that more cells are in M phase. We also observed an increase in cleaved caspase 3 (CCP3) staining following Dyrk1a inhibition (Fig. 4J-L, *P*<0.0001), or following MO or CRISPR injection (Fig. S4A,B). These results generate hypotheses about the apparent paradox between a smaller brain (Fig. 2) and an increase in markers of proliferation (Fig. 3). It is possible that stalling in M phase, perhaps through disruption of the mitotic spindle, could increase the number of apparently proliferative cells, yet lead to a decrease in forebrain size, especially if stalling induces apoptosis. Alternatively, rapid progression into S phase could underlie the increase in M phase cells and cell death. Regardless, these data point to a clear role for Dyrk1a in cell cycle progression and survival during *Xenopus* neurogenesis.

In summary, this work reveals a novel role for Dyrk1a in embryonic ciliogenesis and a putative novel role at mitotic spindles during mitosis. Both processes require dynamic microtubule remodeling, and Dyrk1a has been shown to directly regulate microtubule dynamics during dendrite development (Ori-McKenney et al., 2016). Therefore, we hypothesize that the ciliogenesis and cell cycle phenotypes observed following loss of Dyrk1a both arise from an underlying deficit in microtubule dynamics. Future work should directly explore the role of Dyrk1a in mitotic spindle function. It should also determine whether cells are stalled in M phase rather than transitioning quickly from G1 to S, thereby resulting in an increase in the number of M-phase cells. It is also important to understand the mechanism underlying cell death, as well as its relative contribution to the observed small forebrain phenotype, compared with cell cycle progression defects. Furthermore, the observed defects in ciliogenesis suggest that potential disruptions of basal bodies and primary cilia should be investigated, as well as concomitant changes in patterning.

Overall, these findings provide insight into the pathobiology underlying *DYRK1A* haploinsufficiency. The identification of conserved phenotypes (microcephaly) between human *DYRK1A* haploinsufficiency and *Xenopus dyrk1a* loss of function reinforces the utility of this model organism for studying developmental disorder risk genes being identified by large-scale genetics efforts (Willsey et al., 2018a; Satterstrom et al., 2020), and informs current studies testing DYRK1A kinase inhibitors as potential therapeutic agents for Down syndrome and Alzheimer's disease (Branca et al., 2017; Stotani et al., 2016; Feki and Hibaoui, 2018; Duchon and Herault, 2016; Neumann et al., 2018), as it predicts side effects in ciliated and proliferative tissues. This work also suggests that the congenital kidney and urogenital tract abnormalities in individuals with *DYRK1A* haploinsufficiency may be due to underlying ciliogenesis defects and that these patients should be carefully evaluated for other cilia-related disorders, such as congenital heart disease, hydrocephalus and retinal degeneration.

## MATERIALS AND METHODS

### *Xenopus* husbandry

*Xenopus tropicalis* adult animals were maintained and cared for according to established IACUC protocols. Animals were wild type and both sexes were used. Animals were ovulated using human chorionic gonadotropin (Sigma) according to Sive et al. (2000), and both *in vitro* fertilizations and natural matings were used. Embryos were staged according to Nieuwkoop and Faber (1958).

### Whole-mount RNA *in situ* hybridization

*X. tropicalis dyrk1a* cDNA IMAGE clone 7687837 (Morin et al., 2006) and digoxygenin-11-UTP were used to synthesize antisense probe according to standard protocol using SalI restriction enzyme and T7 polymerase (Sive et al., 2007). Embryos were fixed and stained according to a standard protocol (Harland, 1991), with the omission of the proteinase K step when assaying expression in the multiciliated cells on the epidermis.

### Plasmids and cloning

Full-length human *DYRK1A* cDNA (transcript NM_001396) was obtained from the Harvard Plasmid repository (HsCD00082867) and cloned into pDONR221. LR cloning reactions added a N-terminal GFP using destination vector *pCS-EGFP*, a kind gift from John Wallingford (University of Texas at Austin, USA) (Tu et al., 2018). Sanger sequencing verified the sequences (ElimBio). *Centrin-CFP* was a kind gift from Peter Walentek (University of Freiburg, Germany) (Walentek et al., 2016; Antoniades et al., 2014; Park et al., 2008).

### Embryonic injections

Embryos were injected with 2 nl per blastomere into one cell at the two-cell stage using a Narishige micromanipulator and a Parker Picospritzer III. Plasmid (*hDYRK1A-GFP*) was injected at 40 pg per embryo and mRNA (*Centrin-CFP*) was injected at 500 pg per embryo. Morpholino was injected at 2 ng per embryo; sgRNA was injected at 800 pg per embryo; and Cas9-NLS protein (MacroLabs, UC Berkeley; Lingeman et al., 2017) was injected at 1.5 ng per embryo. Fluorescent dextrans were co-injected to label the injected side of the embryo and animals were sorted left from right-injected at neurula stages.

### Fluorescence staining

Immunostaining was carried out according to Willsey et al. (2018b), with the omission of the bleaching step whenever phalloidin was included. Phalloidin was added during the secondary antibody incubation to visualize actin (1:50, Life Technologies A12380 or A22287). Mounting media with DAPI was used to visualize DNA (Vectashield, Fisher NC9524612). The following primary antibodies were used: DYRK1A (1:100, Abcam, ab69811), acetylated α-Tubulin (1:700, Sigma, T6793), β-Tubulin (1:100, DSHB, clone E7), PCNA (1:50, Life Technologies, clone PC10), polyglutamylated Tubulin (1:100, AdipoGen, GT335), phospho-histone H3 (1:100, Ser10, Sigma, 06-570) and cleaved caspase 3 (1:100, Asp175, Cell Signaling, 9661). Secondary fluorescence-conjugated antibodies were used at 1:250 (Life Technologies, A32723 and A32732).

### *dyrk1a* loss of function

Published and validated translation-blocking *dyrk1a* morpholino (5′ TGCATCGTCCTCTTTCAAGTCTCAT 3′) (Blackburn et al., 2019) was injected at 2 ng per embryo. sgRNA against *X. tropicalis dyrk1a* was designed using CRISPRscan (Moreno-Mateos et al., 2015) (target sequence: 5′-CGTTTAGGTTCTGCTGACGGCGG-3′, oligo sequence: 5′-taatacgactcactataGGTTTAGGTTCTGCTGACGGgttttagagctagaa-3′) and was synthesized *in vitro* (Engen) and purified (Zymo). Purified Cas9-NLS protein was acquired from MacroLab (UC Berkeley) (Lingeman, Jeans, and Corn, 2017). For genotyping, genomic DNA was isolated and the region around the protospacer adjacent motif (PAM) site was amplified by PCR using primers (F: 5′-GGAGAAATCCCTGACAATTGTATTAATTATAGCATTG-3′ and R: 5′-GTTCTTGACCGGTACTGACAAAATGAG-3′) and Sanger sequenced. Mutational efficiency was estimated by sequence deconvolution by tracking of INDELs (TIDE) (Brinkman et al., 2014; DeLay et al., 2018).

### Drug treatment

DYRK1A inhibitor harmine (Sigma Aldrich, 286044) (Göckler et al., 2009; Bain et al., 2007) was reconstituted in dimethylsulfoxide (DMSO) as a 1 mM stock solution. Animals were treated with 1.25 µM of harmine in 1/9 modified ringers or an equal volume of DMSO in 1/9 modified ringers at stage 30, unless otherwise indicated. The embryos were raised until fixation at stage 46 for immunofluorescence staining. Culture liquid was not refreshed during treatment for either condition. Dead animals were removed as soon as they were observed.

### Microscopy

RNA *in situ* hybridization embryos were visualized on a Zeiss AxioZoom.V16 with a 1× objective and a Zeiss 105 color camera with extended depth of focus. Localization and cilia images were acquired on a Leica SP8 laser scanning confocal with a 63× objective. Tadpole nervous system images were acquired by Zeiss AxioZoom.V16 with a 1× objective with a Zeiss 506 monochrome camera and apotome. Whole-mount images are maximum intensity projections of optical sections. We have previously shown that our antibody staining protocol penetrates the brain and that this imaging strategy is sufficient to detect the same gross size changes as physical transverse sections (Willsey et al., 2018b).

### Image analyses and statistical analyses

Images were processed in FIJI (NIH) and compiled in Illustrator (Adobe). Differences in area were measured in FIJI using the free-hand selection function followed by the measure function. Cells with positive antibody staining were marked and counted manually in FIJI. Differences in mean area or cell number were tested for statistical significance by non-parametric Mann–Whitney rank sum test (GraphPad Prism 8).

### RNA extraction, library preparation and sequencing

Individual brains from control and bilaterally injected *dyrk1a* CRISPR tadpoles were dissected at stage 46 and immediately put in 200 µl of cold Trizol (Thermo Fisher), pipetted with a 30 gauge needle to dissociate and frozen at −80°C. Carcasses were genotyped to determine mutational efficiency, and the nine most mutagenized brains were selected and pooled into three independent replicates (three brains per replicate). Nine uninjected brains were pooled into three independent replicates (three brains per replicate). RNA extraction, polyA selection and low-input library preparation (500 bp size selection) were performed by the Functional Genomics Laboratory (UC Berkeley). Samples were processed together, barcoded and spread across a sequencing lane to reduce batch effects. Unstranded, 150 paired end sequencing was performed on an Illumina HiSeq 4000 by the Genomics Sequencing Laboratory (UC Berkeley).

### Transcriptome analysis

RNA-seq reads were aligned to the XenBase *X. tropicalis* v9.1 reference genome using STAR v2.7.3 (Dobin et al., 2013) in gene annotation mode with default parameters. Read counts were converted into counts per million (cpm) and genes with more than 1 cpm in at least three samples were retained for differential gene expression (DEX) analysis. Filtered genes were tested for differential expression using DESeq2 v1.24.0 (Love, Huber, and Anders, 2014) with the shrinkage estimator apeglm (Zhu, Ibrahim, and Love, 2019). *P*-values were corrected for multiple testing using the Benjamini and Hochberg FDR correction. Significantly DEX genes are genes that pass a 0.05 significant threshold. Gene Ontology (GO) enrichment analysis of all annotated DEX genes (*n*=221) was performed using the online classification tool PANTHER (Mi et al., 2010) against the available *Xenopus* database using all expressed genes as a background (*n*=15,079).

Because DYRK1A has been shown to phosphorylate splicing factors (de Graaf et al., 2006; Shi et al., 2008), we also analyzed the data to identify differentially expressed exons. DEXseq (Anders, Reyes, and Huber, 2012) with default parameters was used to quantify exon-level counts and subsequently identify differential exon usage between wild-type and *dyrk1a* knockdown samples. Genes with total exon-level counts below 5 were removed before differential exon usage (DEU) analysis. *P*-values per exon were corrected for multiple testing using the Benjamini and Hochberg FDR correction. There were 97 identified DEU sites within 82 genes which passed a 0.05 significance threshold (Table S3).

## Supplementary Material

10.1242/develop.189290_sup1 Supplementary information
