# Correction: The neurodevelopmental disorder risk gene DYRK1A is required for ciliogenesis and control of brain size in *Xenopus* embryos

**DOI:** 10.1242/dev.198317

**Published:** 2020-12-07

**Authors:** Helen Rankin Willsey, Yuxiao Xu, Amanda Everitt, Jeanselle Dea, Cameron R. T. Exner, A. Jeremy Willsey, Matthew W. State, Richard M. Harland

**Affiliations:** 1 Department of Psychiatry and Behavioral Sciences, Langley Porter Psychiatric Institute, Quantitative Biosciences Institute, and Weill Institute for Neurosciences, University of California San Francisco, San Francisco, CA 94143, USA.; 2 Department of Psychiatry and Behavioral Sciences, Institute for Neurodegenerative Diseases, Quantitative Biosciences Institute, and Weill Institute for Neurosciences, University of California San Francisco, San Francisco, CA 94143, USA.; 3 Department of Psychiatry and Behavioral Sciences, Institute for Neurodegenerative Diseases, Quantitative Biosciences Institute, and Weill Institute for Neurosciences, University of California San Francisco, San Francisco, CA 94143, USA.

There was an error in *Development* (2020) **147**, dev189290 (doi:10.1242/dev.189290).

In the author affiliations, affiliation 2 was incorrect.

**Corrected:**

^1^Department of Psychiatry and Behavioral Sciences, Langley Porter Psychiatric Institute, Quantitative Biosciences Institute, and Weill Institute for Neurosciences, University of California San Francisco, San Francisco, CA 94143, USA.

^2^Department of Molecular and Cell Biology, University of California, Berkeley, Berkeley, CA 94720, USA.

^3^Department of Psychiatry and Behavioral Sciences, Institute for Neurodegenerative Diseases, Quantitative Biosciences Institute, and Weill Institute for Neurosciences, University of California San Francisco, San Francisco, CA 94143, USA.

**Original:**

^1^Department of Psychiatry and Behavioral Sciences, Langley Porter Psychiatric Institute, Quantitative Biosciences Institute, and Weill Institute for Neurosciences, University of California San Francisco, San Francisco, CA 94143, USA.

^2^Department of Psychiatry and Behavioral Sciences, Institute for Neurodegenerative Diseases, Quantitative Biosciences Institute, and Weill Institute for Neurosciences, University of California San Francisco, San Francisco, CA 94143, USA.

^3^Department of Psychiatry and Behavioral Sciences, Institute for Neurodegenerative Diseases, Quantitative Biosciences Institute, and Weill Institute for Neurosciences, University of California San Francisco, San Francisco, CA 94143, USA.

Both the online full-text and PDF versions have been updated.

The authors apologise to readers for this error and any inconvenience this may have caused.

