# Supplementary material for: The neurodevelopmental disorder risk gene DYRK1A is required for ciliogenesis and control of brain size in Xenopus embryos
Source: Development. 2020 Jun 22;147(21):dev189290. doi: 10.1242/dev.189290 (PMC10755402; doi:10.1242/dev.189290)
Supplement: Supplementary information [file develop-147-189290-s1.pdf]

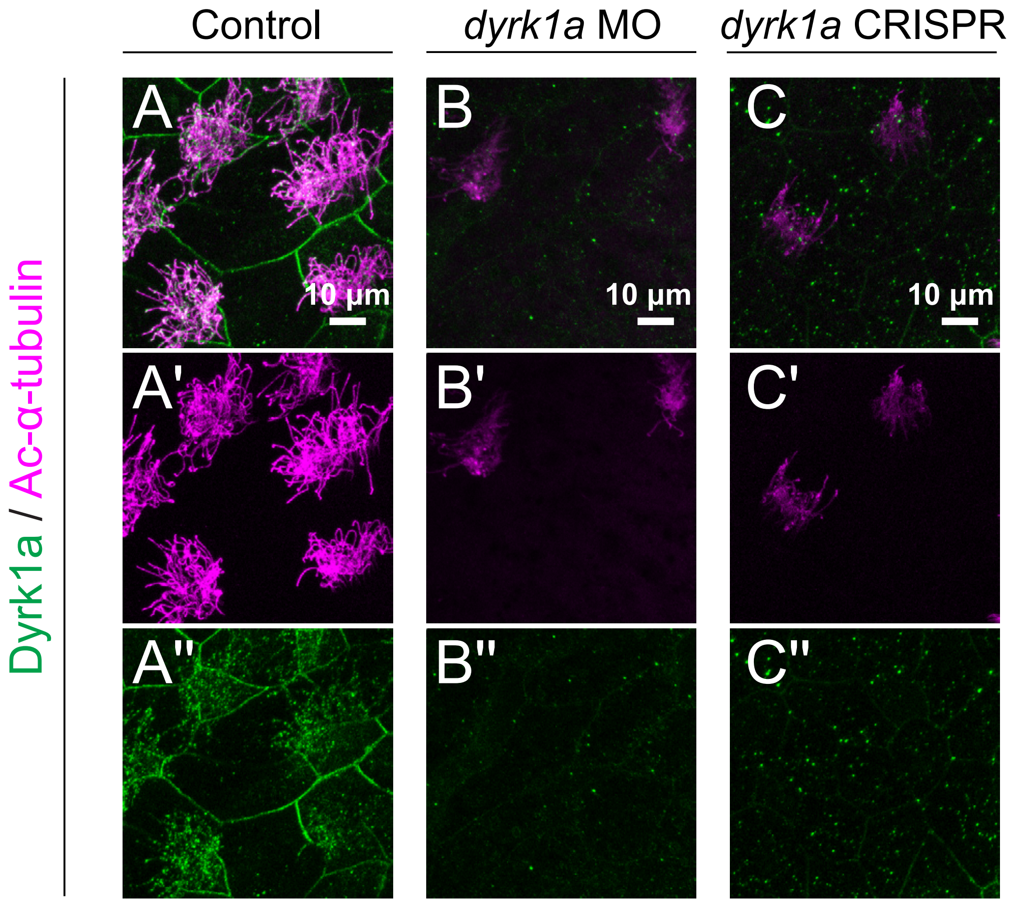

Figure S1: Dyrk1a antibody staining is reduced upon *dyrk1a* perturbation.

(A-C): Acetylated α-Tubulin antibody staining (magenta) and Dyrk1a antibody staining (green) of stage 35 *X. tropicalis* embryonic epidermis. (A) Control injection shows Dyrk1a antibody staining along ciliary axonemes. *dyrk1a* morpholino (MO) (B) or *dyrk1a* CRISPR/Cas9 (C) injection reduces Dyrk1a antibody staining.

Development • Supplementary information

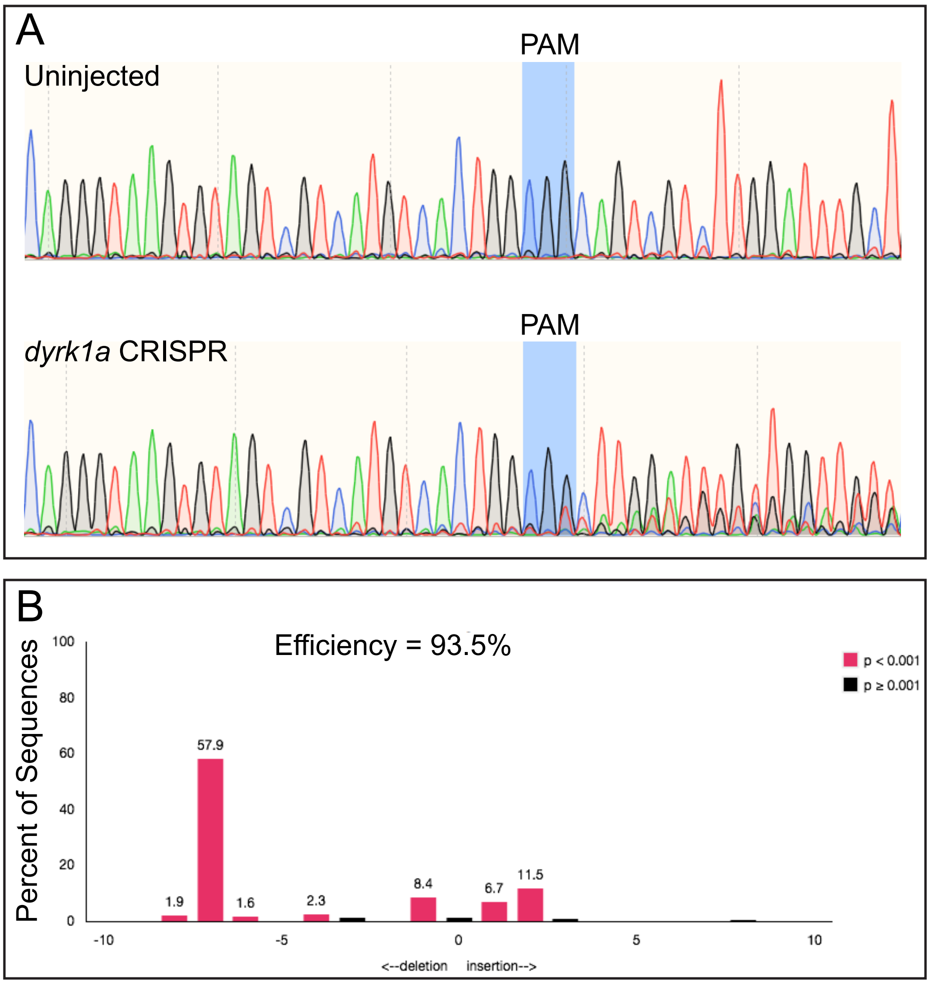

Figure S2: *dyrk1a* CRISPR genotyping.

(A) Examples of Sanger sequencing traces from PCR products amplifying the region surrounding the protospacer adjacent motif (PAM) within the *dyrk1a* coding sequence for uninjected control and *dyrk1a* CRISPR/Cas9 bilaterally injected embryos. Note the drop-off in sequence quality following the PAM, indicating insertions and/or deletions. (B) Example of tracking of INDELs (TIDE) analysis from the injected animal in (A), where Sanger sequencing traces are deconvolved to determine frequency and position of induced variants. For this sample, mutational efficiency is 93.5%, and the frequency and position of insertion and deletion frequency are shown as a histogram. For this sample, the most common sequence was a -7 base pair deletion (57.9% of sequences).

Development • Supplementary information

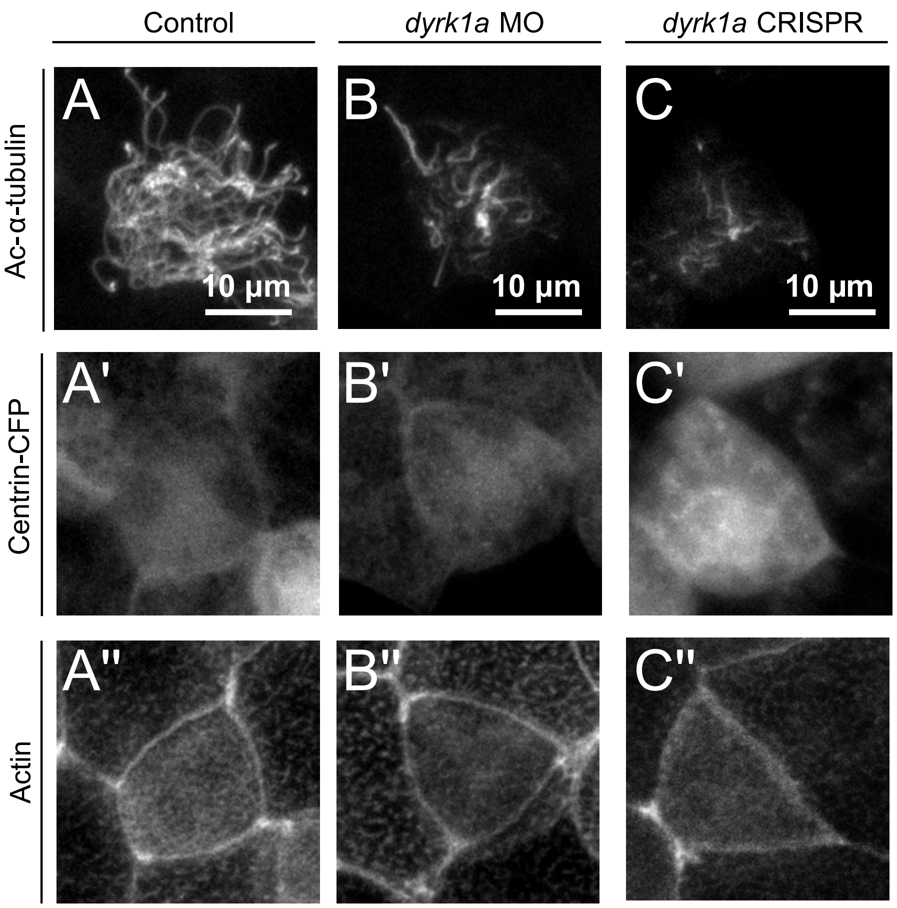

Figure S3: *dyrk1a* is required for ciliogenesis.

(A-C) Separated channels in grayscale from stage 35 *X. tropicalis* embryonic epidermis from Fig. 2. Injection of *dyrk1a* morpholino (MO) (B) or CRISPR/Cas9 reagents (C) causes defects in ciliogenesis compared to the injected control (A).

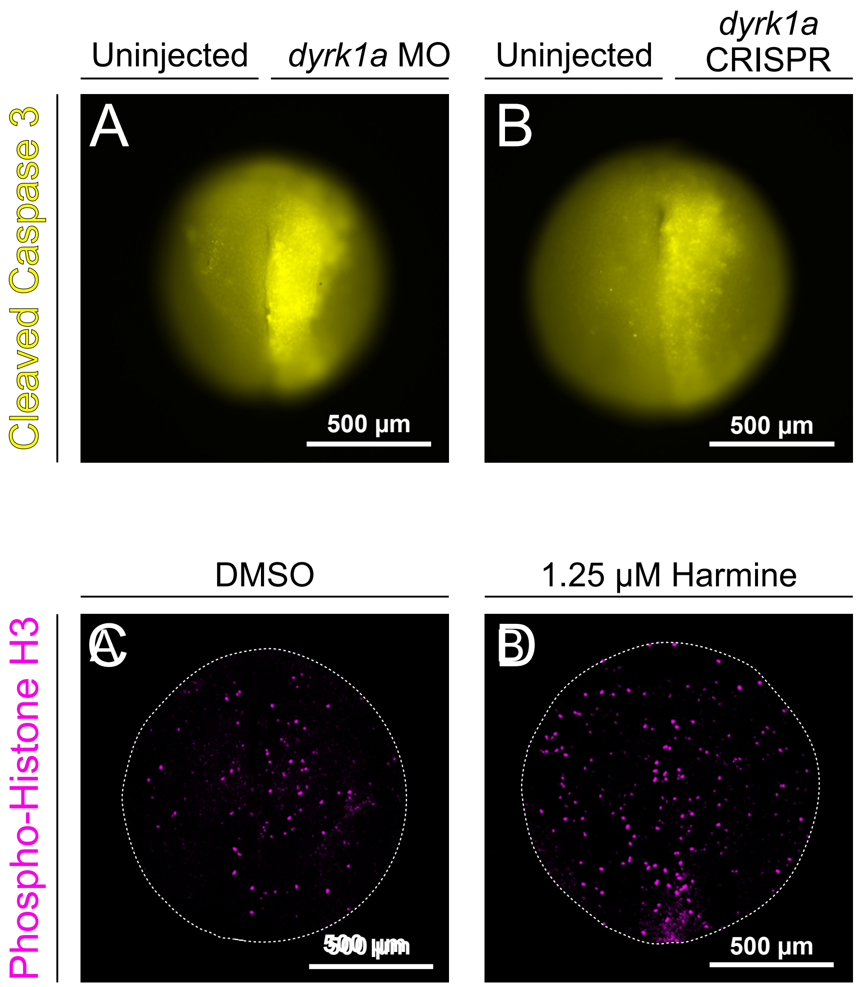

Figure S4: Loss of *dyrk1a* increases cleaved caspase 3 and phospho-histone H3 staining. (A-B) Representative images of cleaved caspase 3 (yellow) staining of *X. tropicalis* neurula stage embryos injected only on the right side with *dyrk1a* morpholino (MO) (A) or *dyrk1a* CRISPR/Cas9 reagents (B). (C-D) Representative images of phospho-histone H3 (magenta) staining of *X. tropicalis* neurula stage embryos treated with DMSO (C) or 10 µM Harmine (D) beginning at blastula stages. Outlines of the embryos are shown in white dashed lines (C-D). All images are dorsal view with the anterior oriented to the top, and maximum intensity projections of optical sections. All conditions have a sample size greater than 20.

Development • Supplementary information

Table S1: Differentially expressed genes following *dyrk1a* CRISPR injection.

Xenopus tropicalis gene symbol, log2 fold change, adjusted p-value (padj), and additional information for 294 genes differentially expressed in stage 46 brains following *dyrk1a* CRISPR injection.

Click here to Download Table S1

Table S2: Enriched gene ontology terms from differentially expressed genes following *dyrk1a* CRISPR injection.

Enriched gene ontology terms, fold enrichment, false discovery rate, and differentially expressed (DEX) genes comprising each category for the 221 annotated genes DEX following *dyrk1a* CRISPR injection.

Click here to Download Table S2

Table S3: Differential exon usage following *dyrk1a* CRISPR injection.

Xenopus tropicalis gene symbol, transcript name, exon number, log2 fold change, adjusted p-value (padj), and additional information for the 97 sites of differential exon usage following *dyrk1a* CRISPR injection.

Click here to Download Table S3
