# Supplementary material for: The neurodevelopmental disorder risk gene DYRK1A is required for ciliogenesis and control of brain size in Xenopus embryos
Source: Development. 2020 Jun 22;147(21):dev189290. doi: 10.1242/dev.189290 (PMC10755402; doi:10.1242/dev.189290)
Supplement: Supplementary file 2 [file develop-147-189290_review_history.pdf]

Neurodevelopmental disorder risk gene *DYRK1A* is required for ciliogenesis and control of brain size in *Xenopus* embryos

Helen Rankin Willsey, Yuxiao Xu, Amanda Everitt, Jeanselle Dea, Cameron R.T. Exner, A. Jeremy Willsey, Matthew W. State and Richard M. Harland
DOI: 10.1242/dev.189290

Editor: John B. Wallingford

Review timeline

Original submission:	10 February 2020
Editorial decision:	5 March 2020
First revision received:	29 April 2020
Accepted:	11 May 2020

Original submission

First decision letter

MS ID#: DEVELOP/2020/189290

MS TITLE: Neurodevelopmental disorder risk gene DYRK1A is required for ciliogenesis and brain size control in Xenopus embryos

AUTHORS: Helen Rankin Willsey, Yuxiao Xu, Amanda Everitt, Jeanselle Dea, Cameron R.T. Exner, A. Jeremy Willsey, Matthew W. State, and Richard M. Harland

I have now received all the referees' reports on the above manuscript, and have reached a decision. The referees' comments are appended below, or you can access them online: please go to BenchPress and click on the 'Manuscripts with Decisions' queue in the Author Area.

As you will see, the referees express considerable interest in your work, but have some significant criticisms and recommend a substantial revision of your manuscript before we can consider publication.

As you will also see, no one major issue stood out for all three reviewers,so while i'd ask that you carefully consider all of these reviews, it is not reasonable to expect that you would make all of recommended addition. Therefore, i'd like for you to take a look at the reviews andand then get back to me with what you feel is a reasonable action plan for revision. The plan should focus first on overcoming the technical concerns of the reviewers, and secondarily on slightly enlarging the scope, as suggested in various ways, especially by reviewers #1 and #3.

If you are able to revise the manuscript along the lines suggested, which may involve further experiments, I will be happy receive a revised version of the manuscript. Your revised paper will be re-reviewed by one or more of the original referees, and acceptance of your manuscript will depend on your addressing satisfactorily the reviewers' major concerns. Please also note that Development will normally permit only one round of major revision.

Please attend to all of the reviewers' comments and ensure that you clearly highlight all changes made in the revised manuscript. Please avoid using 'Tracked changes' in Word files as these are lost in PDF conversion. I should be grateful if you would also provide a point-by-point response detailing

© 2020. Published by The Company of Biologists under the terms of the Creative Commons Attribution License (https://creativecommons.org/licenses/by/4.0/).

how you have dealt with the points raised by the reviewers in the 'Response to Reviewers' box. If you do not agree with any of their criticisms or suggestions please explain clearly why this is so.

Reviewer 1

Advance summary and potential significance to field

The authors convincingly define a novel role of DYRK1A for regulation of ciliogenesis and brain size control in Xenopus. They show that DYRK1A is expressed in ciliated tissues and the brain and demonstrate subcellular localization within the ciliary axonemes and at basal bodies of the Xenopus multiciliated cells of the epidermis and localizes to the nucleus. Using both CRISPR knockout and morpholino reagents for loss of function studies, they describe a dual role for DYRK1A in ciliogenesis and brain size during development.

Transcriptomic analysis of dyrk1a crispants suggesting that DYRK1A controls brain size by controlling progression through the cell cycle. Finally, by modifying the localization of DYRK1A into cytoplasmic or nuclear regions, the authors show that DYRK1A function in the cytoplasm is critical for its role in brain size control. Although the authors provide several pieces of convincing evidence supporting their conclusions and well written manuscript, there are some major concerns that would need to be addressed before publication in Development.

Comments for the author

I would encourage a revision of this manuscript to highlight key molecular changes involving alterations in brain development in their model.

Major concerns:

1. In contralateral injections the authors show that in both dyrk1a morphants and CRISPR-targeted injected embryos (crispants) develop smaller telencephalons. The authors then go on to suggest that this is through the cell cycle control function of DYRK1A, concluding that increased proliferation by whole-mount staining of PCNA leads to reduced brain size. This conclusion should be greatly bolstered by analysis of the telencephalon and other parts of the brain in fixed sections, as the staining pattern in whole-mount is not very informative nor easily interpretable. Moreover, a better description of the proliferative changes in the context of cortex differentiation in a multiple brain region would greatly improve our understanding of the regulation of Dyrk1a for brain development. This should be done for: (i) the pharmacological inhibitor; and (ii) either the morphant or the crispant phenotypes.

2. A key question for me is whether the patterning of the brain is affected? This should be assayed with Nissl stained sections of multiple brain regions in all experimental conditions outlined above. Moreover, are primary cilia affected in the brain?

3. If proliferation is increased then why is macrocephaly not observed? An obvious analysis to address this is to assay markers of cell death (cleaved Caspace 3) on fixed sections in variety of brain regions.

4. Rescue experiments using the nuclear export or nuclear import constructs were never assayed in the context of the cell cycle defect. Analysis of these molecular localization rescue experiments should be analyzed in fixed sections using markers of proliferation, cell death, and for assay of cortex development.

5. Another big question I was left with was whether the synthetic dykr1a-NES RNA would rescue the ciliogenesis defects in the ciliated epidermis in crispants for instance? Is the proliferation status affected in the multiciliated cells in the epidermis in crispants?

6. Are the basal bodies affected in the dyrk1a morphants or crispants? The centrin-BFP is barely perceptible in the Figure 2. Possibly a different pseudocolor scheme would make this signal stand out more. I would recommend putting the three channels in grey scale below the merged image to really allow the reader to see each independently.

© 2020. Published by The Company of Biologists under the terms of the Creative Commons Attribution License (https://creativecommons.org/licenses/by/4.0/).

Minor concerns:
1. Given the DYRK1A is associated with Trisomy 21/Downs' Syndrome. Is there any evidence that a gain-of-function contributes to a phenotype in your model?

2. The authors show that DYRK1A is associated with the basal bodies and axonemes in multiciliated cells. The staining pattern with antibody is punctate along the axonemes, reminiscent of IFT and trafficking within the axoneme. Is DYRK1A stably associating with the axonemes in multiciliated cells or is it trafficking along the axonemes? Live imaging of the synthetic dkyk1a-GFP construct would easily define its motility within the axoneme.

3. The in-situ showing ciliated epidermal expression is not convincing, possibly a higher magnification of the epidermis showing the expression pattern is warranted. Not a hill worth dying however, as it is clear that DYRK1a is expressed in ciliated cells by the antibody and synthetic fluorescently tagged mRNA expression in these cells.

4. Do the dyrk1a targeting CRISPR and morpholino reagents affect the nuclear pool of DYRK1A?? The authors show efficient loss of DYRK1A antibody staining in the ciliated epidermis; however, they do not show if this is true for the nuclear pool of DYRK1A. Including this information is important to how effective the CRISPR and morpholino are in disrupting DYRK1A function.

5. Do all cells have a nuclear pool of DYRK1A? Please clarify if it is known.

6. The embryo in Figure 1B is dorsal to the random and not really a good lateral view, please rotate to conserve a standard orientation of all animals throughout the paper. Possibly redo this in situ with a less bend embryo, with an inset of the ciliated epidermis?

7. I don't think these experimental animals should be called mutants. I would define them as morphants or crispants to avoid confusion, and to be honest about there chimeric nature. That said this experiment should be judged with the same reference as a germline mutant given the care of these well controlled experiments.

8. Missed a few Greek letters for alpha tubulin.

<u>Reviewer 2</u>

Advance summary and potential significance to field

The manuscript by Willsey at al. uncovers a novel role for Dyrk1a in ciliogenesis and establishes a model for microencephaly in a non-mammalian model. The authors identify potentially dysregulated pathways and a hyperproliferation phenotype upon chemical inhibition. Rescues with constructs that alter the nuclear localization behavior of Dyrk1a suggest a surprising role outside of the nucleus. The paper addresses a clinically relevant issue and describes an unexpected, extranuclear mode of action. Each of the findings is interesting, but not all are necessarily linked. However, this new disease model is of great value to structural brain disorder research.

Comments for the author

The connection between the ciliary phenotype and an effect on cell cycle control in brain development are rather speculative. Can any additional evidence strengthen this intriguing connection?

"expression is highest in highly ciliated tissues including the epidermis"
It is curious that the in situ does not show an epidermal spotted pattern typical for multi-ciliated cells. I appreciate the knockdown control for the immunostaining in epidermal ciliated cells. The predominantly stained tissues are not particularly cilia rich. I don't fully follow the argument that this expression pattern clearly points to a ciliary enrichment. Has the specificity of the antibody been validated using a CRISPR or Mo control also on the strongly staining neural tissue? Is the nuclear localization also detectable by immunostaining or only visible by overexpression?

© 2020. Published by The Company of Biologists under the terms of the Creative Commons Attribution License (https://creativecommons.org/licenses/by/4.0/).

To me there seems to be a signal at the cell membranes, please comment.

Dyrk1a is “localized to microtubules of ciliary axonemes” – this statement is not sufficiently supported by evidence. The protein might as well reside in the ciliary membrane or be a component of ciliary cargo complexes. Do the authors have additional evidence for a microtubular localization?

Fig 1I olfactory epithelium – I can (squirting my eyes) see the ciliary localization, but isn’t the majority of protein found in lower layers (maybe membranes again?) Please comment. If this is such a prominently expressing area, does the knockdown effect its morphology?

The ciliary defect is striking, but should be quantified. Are cilia of ependymal cells nicely shown in figure 1J also effected by the knockdown? Or are any primary cilia in neuronal cells or elsewhere shortened? It’s worth to know if there is a general or tissue specific ciliogenesis defect.

The RNA-Seq read alignment show that CRISPR induced indels at the targeted locus, but is there any evidence for nonsense mediated decay of the dyrk1a mRNA?
It does not seem to appear in the volcano plot. Is it possible that alternative initiation sites provide a read-through mechanism that doesn’t much alter mRNA and protein levels? This may explain the relatively modest effect on brain size as compared to the MO. Is there any evidence that spicing events in general are altered, as this is a proposed canonical function of Dyrk1a?

What is the proportion of the differentially regulated genes predominantly expressed in neuronal tissue? Are disease related genes among the regulated transcripts? It might be worth doing the GO analysis separately for up- or down-regulated transcripts.

Fig 3E: What is the reason for leaving the previously validated genetic approach at this point and switch to pharmacological Dyrk1a inhibition? Is total brain size reduced in presence of the inhibitor as shown for the Mo/Crispr situation? Is PCNA staining increases on the Mo/Crispr injected side? Are there any know targets of Dyrk1a that may explain the cell cycle extension from a molecular perspective?

Fig 4: This interesting data deserves rigorous quantification and statistics. Is PCNA staining affected (reduced) by overexpression of Dyrk1a? Does a kinase dead Dyrk1a-NES fail to rescue?
From the methods section, it seems that plasmid DNA was injected, and I would expect a highly mosaic expression. The expression of the constructs should be monitored as the constructs are already GFP tagged.

Fig. S3: The nuclear size seems very different between A and C (scale bar similar) is this just by coincidence or a phenotype?

“Leverage this system to which Dyrk1a’s function are relevant to ASD” – I see that this is a very nice model for structural brain anomalies, in particular microencephalopathy. But how could a psychiatric condition, such as ASD be modelled in tadpoles?

Minor points:

The source of the Cas9-NLS protein should be given.

Fig 2 F: ratios of injected vs. uninjected sides should be shown as log2-fold values.
This will result in equidistant values < and > 1.

Reviewer 3

Advance summary and potential significance to field

This efficient study brings compelling evidence that dyrk1a is required for ciliogenesis, an important observation that was missed in human, mouse and Drosophila studies.

© 2020. Published by The Company of Biologists under the terms of the Creative Commons Attribution License (https://creativecommons.org/licenses/by/4.0/).

Comments for the author

This is a succinct yet convincing study on the role played by kinase dyrk1a in the context of Xenopus development. The authors intended, and succeed, to bring forth a novel pathomechanism for DYRK1A haploinsufficiency in humans which is associated with AUTOSOMAL DOMINANT (MIM614104) MENTAL RETARDATION 7 (MR7).

The techniques employed to perform loss-of-function and rescue experiments in Xenopus tropicalis are adequate and the evidence brought forth about the localization of endogenous DYRK1A to cilia are solid.

Of course, it goes without mentioning but an acute depletion or permanent deletion of of DYRK1A in a human cell line capable of ciliogenesis would be a welcome addition to validate the main findings from Xenopus embryos. this would significantly broaden the impact and reach of the conclusions that MR7 is a congenital syndromic ciliopathy.

Besides this I have no major objections to its publication and suggest a couple of experiments that will strengthen the paper.

1. What is the maternal contribution for DYRK1A. Can the other provide a developmental QPCR to document the onset of transcription.

2. Being an established kinase, one would think that that a differential proteome performed on enriched phosphorylated proteins would have been a more accurate readout of the loss of DYRK1A. Instead authors chose to perform an RNAseq which is fine but is reflective of more indirect consequences. In this regard, the top 10 dysregulated genes (e.g. cfd, apoe npas4) found by RNAseq must be validated by QPCR and /or WISH.

3. What is the consequence of the loss of DYRK1A on motile beating cilia.
Can their movement, or lack thereof, be documented. A simple dye assay should suffice.

First revision

Author response to reviewers' comments

Point-by-Point Response, *Willsey & Xu, et al.*, *"Neurodevelopmental disorder risk gene* DYRK1A *is required for ciliogenesis and brain size control in* Xenopus *embryos"*

Reviewer 1 Advance Summary and Potential Significance to Field:
The authors convincingly define a novel role of DYRK1A for regulation of ciliogenesis and brain size control in Xenopus. They show that DYRK1A is expressed in ciliated tissues and the brain and demonstrate subcellular localization within the ciliary axonemes and at basal bodies of the Xenopus multiciliated cells of the epidermis and localizes to the nucleus. Using both CRISPR knockout and morpholino reagents for loss of function studies, they describe a dual role for DYRK1A in ciliogenesis and brain size during development.

Transcriptomic analysis of dyrk1a crispants suggesting that DYRK1A controls brain size by controlling progression through the cell cycle. Finally, by modifying the localization of DYRK1A into cytoplasmic or nuclear regions, the authors show that DYRK1A function in the cytoplasm is critical for its role in brain size control. Although the authors provide several pieces of convincing evidence supporting their conclusions and well written manuscript, there are some major concerns that would need to be addressed before publication in Development.

Reviewer 1 Comments for the Author:
I would encourage a revision of this manuscript to highlight key molecular changes involving alterations in brain development in their model.

© 2020. Published by The Company of Biologists under the terms of the Creative Commons Attribution License (https://creativecommons.org/licenses/by/4.0/).

We thank the reviewer for their careful reading and helpful criticism. We have now revised the manuscript to include an entirely new figure (Fig. 4), which focuses on characterizing the role of Dyrk1a in controlling cell cycle progression, differentiation, and cell survival in the developing brain.

Major concerns:

1. In contralateral injections the authors show that in both dyrk1a morphants and CRISPR-targeted injected embryos (crispants) develop smaller telencephalons. The authors then go on to suggest that this is through the cell cycle control function of DYRK1A, concluding that increased proliferation by whole-mount staining of PCNA leads to reduced brain size. This conclusion should be greatly bolstered by analysis of the telencephalon and other parts of the brain in fixed sections, as the staining pattern in whole-mount is not very informative nor easily interpretable. Moreover, a better description of the proliferative changes in the context of cortex differentiation in a multiple brain region would greatly improve our understanding of the regulation of Dyrk1a for brain development. This should be done for: (i) the pharmacological inhibitor; and (ii) either the morphant or the crispant phenotypes.

Our images of whole-mount stainings are maximum intensity projections of optical sections. We have previously shown that our whole-mount staining protocol penetrates the entire tadpole brain (*Willsey et al, 2018 Dev. Bio.*), and that the conclusions drawn from our whole-mount imaging of brain size and gross anatomy recapitulate the conclusions made from physical transverse sections (See Fig. 7 of *Willsey et al, 2018 Dev. Bio.*). We have now added this clarification to our methods section (Lines 271-274).

With respect to other brain regions, we chose to focus on the telencephalon in this manuscript because (1) we observe the strongest size phenotype when comparing this region between crispants ($p < 0.01$ for forebrain versus $p > 0.05$ for midbrain and hindbrain); and (2) *DYRK1A* is a high confidence ASD gene that converges with other ASD genes to form a nexus of risk in developing human forebrain (*Willsey, et al., Cell 2013*).

To address the request for further analysis of proliferation and differentiation, we now show analyses of 2 markers of proliferation (PCNA and phospho-histone H3) to assay S and M phases, respectively (Fig. 4). Pharmacological inhibition of Dyrk1a results in the same small-brain phenotype as the morphants and crispants (new data; Fig. 2G, I), and therefore, we conducted these analyses using pharmacological inhibition, as this gives us the ability to precisely control the timing of the perturbation, thereby separating cell cycle defects from earlier phenotypes. First, we demonstrated that markers of both S and M phase are increased following Dyrk1a inhibition (Fig. 4C-I). Next we assayed the effect on differentiation by comparing the ratio of NPCs to differentiated neurons, and observed a relative increase in NPCs at the expense of differentiated neurons (Fig. 4F). We also assayed cell death by cleaved caspase 3 (CCP3) staining and show that in all cases (morphants, crispants, and pharmacological inhibition), CCP3 staining is increased following *dyrk1a* inhibition (Fig. 4J-L, Fig. S4A-B). Given the increase in mitotic cyclin B expression in crispants (Fig. 3D) and the localization of Dyrk1a on mitotic spindles (new data; Fig. 4A-B), we now propose that while these cells are positive for classic proliferation markers, they potentially have defects in cell cycle progression and have increased cell death, leading to a smaller brain.

2. A key question for me is whether the patterning of the brain is affected? This should be assayed with Nissl stained sections of multiple brain regions in all experimental conditions outlined above. Moreover, are primary cilia affected in the brain?

While gross patterning (ie, forebrain/midbrain/hindbrain distinctions) does not appear to be affected, we cannot rule out that there are more subtle changes in patterning following Dyrk1a inhibition. While we agree with the reviewer that this is an important question, we chose to focus this manuscript on the telencephalon size phenotype, as it offers a clear window into the biological function of Dyrk1a in *Xenopus* brain development and our RNA-Seq data indicated a cell cycle disruption, not a global patterning defect.

© 2020. Published by The Company of Biologists under the terms of the Creative Commons Attribution License (https://creativecommons.org/licenses/by/4.0/).

Given the disruptions in ciliogenesis in multiciliated cells of the epidermis, one would hypothesize that there would be similar defects in primary cilia. However, we do not yet have evidence for this. Presumably, a loss of primary cilia would result in a loss of hedgehog signaling, and therefore, midline defects and other gross anatomical phenotypes like loss of the thalamus (see image below showing these phenotypes resulting from hedgehog inhibition by classic inhibitor cyclopamine). We do not observe these phenotypes, nor do we see a reduction in hedgehog target genes like *patched1* and *gli1* in the RNA-Seq data. Further, a loss of hedgehog signaling would promote neural differentiation (see below, loss of PCNA+ cells), and instead we see an increase in the relative proportion of neural progenitor cells compared to differentiated cells (Fig. 4F).
Therefore, we think the cell cycle progression defect is the main driver of the brain size reduction and is most relevant to this manuscript. We do however, comment in lines 188- 190 that future work should assay the effect of *dyrk1a* loss on primary cilia and patterning in the brain.

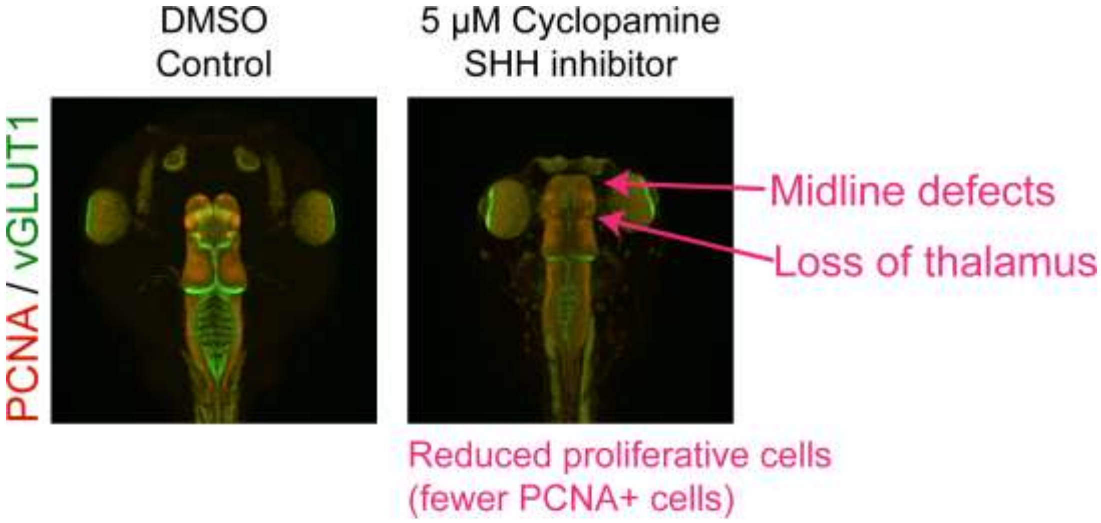

3. If proliferation is increased then why is macrocephaly not observed? An obvious analysis to address this is to assay markers of cell death (cleaved Caspace 3) on fixed sections in variety of brain regions.

We thank the reviewer for this comment and have now included cleaved caspase 3 (CCP3) antibody staining for *dyrk1a* crispants, morphants, and following pharmacological treatment (Fig. 4J-L, Sig. S4A-B). In all cases we observe an increase in CCP3 staining throughout the manipulated tissue, which could explain the apparent paradox between an increase in apparently proliferative cells and the microcephaly observed. Again, however, we would like to emphasize that these 'apparently proliferative cells' may actually be delayed in cell cycle progression, leading to a smaller brain (and perhaps contributing to the increased cell death observed).

4. Rescue experiments using the nuclear export or nuclear import constructs were never assayed in the context of the cell cycle defect. Analysis of these molecular localization rescue experiments should be analyzed in fixed sections using markers of proliferation, cell death, and for assay of cortex development.

Due to concerns from Reviewer 2 on the potential for mosaic expression from injected plasmids, we became concerned about the potential for false negatives from these rescue experiments since we did not assess GFP expression for mosaicism concurrently. Therefore, we have replaced this figure (old Fig. 4) with a dissection of cell cycle progression, differentiation, and cell death following *dyrk1a* depletion instead (new Fig. 4). In the revised manuscript, we now only present rescue by wild type Dyrk1a (i.e. cytoplasmic and nuclear localized) in Fig. 2H-I. As per the reviewers comments, we also added quantification of the rescue.

5. Another big question I was left with was whether the synthetic dykr1a-NES RNA would rescue

© 2020. Published by The Company of Biologists under the terms of the Creative Commons Attribution License (https://creativecommons.org/licenses/by/4.0/).

the ciliogenesis defects in the ciliated epidermis in crispants for instance? Is the proliferation status affected in the multiciliated cells in the epidermis in crispants?

As in point 4, we have decided that mosaic plasmid expression may lead to false negative results, and we have moved away from these kinds of analyses for the time being until we can monitor plasmid GFP expression per cell. Regarding your second point, we assayed proliferation in the epidermis of these earlier stages following Dyrk1a inhibition by phospho-histone H3 staining, and also observed an increase in apparently proliferative cells (Fig. S4C-D).

6. Are the basal bodies affected in the dyrk1a morphants or crispants? The centrin-BFP is barely perceptible in the Figure 2. Possibly a different pseudocolor scheme would make this signal stand out more. I would recommend putting the three channels in grey scale below the merged image to really allow the reader to see each independently.

In our original manuscript, we did not comment about whether basal bodies were affected by *dyrk1a* inhibition, and our only claim was that *dyrk1a* is required for ciliogenesis. In this experiment, we had used Centrin-CFP as an injection marker and to be able to identify multiciliated cells. We now present all the channels in grayscale in Fig. S3. Unfortunately we do not feel comfortable commenting on whether basal bodies are disrupted from these and other images we acquired without being able to rigorously quantify at high resolution. We cannot repeat this experiment at this time due to COVID- 19 restrictions. Instead, we now note that this is an important future direction since Dyrk1a localizes to basal bodies (Lines 188-190).

Minor concerns:
1. Given the DYRK1A is associated with Trisomy 21/Downs' Syndrome. Is there any evidence that a gain-of-function contributes to a phenotype in your model?

This paper is primarily focused on the consequences of *dyrk1a* loss of function. However, we do observe a statistically significant increase in telencephalon size following over-expression of *hDYRK1A-GFP*. We would like to include this experiment in a future manuscript, and we feel that this analysis is not essential for this paper since its primary focus is on *dyrk1a* loss of function.

2. The authors show that DYRK1A is associated with the basal bodies and axonemes in multiciliated cells. The staining pattern with antibody is punctate along the axonemes, reminiscent of IFT and trafficking within the axoneme. Is DYRK1A stably associating with the axonemes in multiciliated cells or is it trafficking along the axonemes? Live imaging of the synthetic dkyk1a-GFP construct would easily define its motility within the axoneme.

While an interesting experiment, we think this kind of analysis would be better suited to a different manuscript on the precise relationship between DYRK1A and microtubules and axonemes, and think that it is not essential for this manuscript.

3. The in-situ showing ciliated epidermal expression is not convincing, possibly a higher magnification of the epidermis showing the expression pattern is warranted. Not a hill worth dying however, as it is clear that DYRK1a is expressed in ciliated cells by the antibody and synthetic fluorescently tagged mRNA expression in these cells.

We have replaced this image and included a high-magnification inset to better show the epidermal RNA *in situ* staining (Fig. 1B-B').

4. Do the dyrk1a targeting CRISPR and morpholino reagents affect the nuclear pool of DYRK1A?? The authors show efficient loss of DYRK1A antibody staining in the ciliated epidermis; however, they do not show if this is true for the nuclear pool of DYRK1A. Including this information is important to how effective the CRISPR and morpholino are in disrupting DYRK1A function.

We do not observe Dyrk1a in the nucleus by antibody staining like we do for over- expression of the human DYRK1A-GFP construct. Therefore, we could not assay whether inhibition reduces this staining. Since we have no longer investigated the role of nuclear Dyrk1a

© 2020. Published by The Company of Biologists under the terms of the Creative Commons Attribution License (https://creativecommons.org/licenses/by/4.0/).

specifically in development, we have removed this localization from the manuscript.

5. Do all cells have a nuclear pool of DYRK1A? Please clarify if it is known.

We do not observe a nuclear pool of Dyrk1a using the antibody on ciliated epidermis. Since we have no longer investigated the role of nuclear Dyrk1a specifically in development, we have removed this localization from the manuscript.

6. The embryo in Figure 1B is dorsal to the random and not really a good lateral view, please rotate to conserve a standard orientation of all animals throughout the paper. Possibly redo this in situ with a less bend embryo, with an inset of the ciliated epidermis?

We have replaced this image with one of a lateral view of a less bent embryo, oriented with dorsal up and anterior to the left (Fig. 1B). We have also included a higher- magnification inset of the ciliated epidermis (Fig. 1B').

7. I don't think these experimental animals should be called mutants. I would define them as morphants or crispants to avoid confusion, and to be honest about there chimeric nature. That said this experiment should be judged with the same reference as a germline mutant given the care of these well controlled experiments.

We appreciate this point and no longer refer to these animals as 'mutants' in the revised manuscript. Rather, we use 'crispant' or 'CRISPR-injected.'

8. Missed a few Greek letters for alpha tubulin.

We have scanned the manuscript and ensure that this has been addressed.

Reviewer 2 Advance Summary and Potential Significance to Field:
The manuscript by Willsey at al. uncovers a novel role for Dyrk1a in ciliogenesis and establishes a model for microencephaly in a non-mammalian model. The authors identify potentially dysregulated pathways and a hyperproliferation phenotype upon chemical inhibition. Rescues with constructs that alter the nuclear localization behavior of Dyrk1a suggest a surprising role outside of the nucleus. The paper addresses a clinically relevant issue and describes an unexpected, extranuclear mode of action. Each of the findings is interesting, but not all are necessarily linked. However, this new disease model is of great value to structural brain disorder research.

Reviewer 2 Comments for the Author:
The connection between the ciliary phenotype and an effect on cell cycle control in brain development are rather speculative. Can any additional evidence strengthen this intriguing connection?

We thank the reviewer for their careful reading of the manuscript, and have added new data suggesting that these two phenotypes may result from a common molecular mechanism--- namely, disruption of microtubule dynamics. Specifically, in the revised manuscript, we now present an entirely new figure (Fig. 4) showing that Dyrk1a also localizes to mitotic spindles (Fig. 4A-B) and loss causes an increase in cells in M phase (Fig. 4G-I) and an induction of cell death (Fig. 4J-L). Therefore, we now pose the hypothesis that Dyrk1a may be important for microtubule dynamics in the context of both ciliogenesis and mitosis, which could result in both of the observed phenotypes (Line 179-183). In line with this idea, Dyrk1a has previously been shown to affect microtubule dynamics by directly phosphorylating β-Tubulin in the context of neuronal dendrite development (Ori-McKenney et al., 2016).

"expression is highest in highly ciliated tissues including the epidermis"

It is curious that the in situ does not show an epidermal spotted pattern typical for multi-ciliated cells. I appreciate the knockdown control for the immunostaining in epidermal ciliated cells. The predominantly stained tissues are not particularly cilia rich. I don't fully follow the argument that this expression pattern clearly points to a ciliary enrichment. Has the specificity of the antibody been validated using a CRISPR or Mo control also on the strongly staining neural tissue? Is the

© 2020. Published by The Company of Biologists under the terms of the Creative Commons Attribution License (https://creativecommons.org/licenses/by/4.0/).

nuclear localization also detectable by immunostaining or only visible by overexpression? To me there seems to be a signal at the cell membranes, please comment.

In the revised manuscript, we now present a new image for the epidermal RNA *in situ* expression with a higher-magnification inset of the ciliated epidermis (Fig. 1B-B'). It is now apparent that *dyrk1a* is expressed in the typical spotted pattern. Additionally, in our experience, seeing strong expression in such a spotted pattern in the epidermis, combined with strong kidney and otic vesicle staining, does often predict a potential role in ciliary biology, which is why we assayed localization in the ciliated epidermis first. Nonetheless, we have rewritten this section to be less strongly worded (Lines 78-86).

The antibody validation experiments in the epidermis show that the antibody is strikingly reliable; therefore we have not directly tested it in other tissues. We feel our original validations are sufficient.

The nuclear localization observed following over-expression of the human GFP-tagged construct was not observed with the antibody for endogenous Dyrk1a, and since we no longer present data related to a nuclear function, we have removed this localization. We agree that there is signal near cell membranes, and in the revised manuscript we address this and show that it is localization of Dyrk1a near sites of α-Tubulin (Fig. 4A-B).

Dyrk1a is "localized to microtubules of ciliary axonemes" – this statement is not sufficiently supported by evidence. The protein might as well reside in the ciliary membrane or be a component of ciliary cargo complexes. Do the authors have additional evidence for a microtubular localization

We have edited this sentence to now read that Dyrk1a "localizes to ciliary axonemes" (Line 93-94).

Fig 1I olfactory epithelium – I can (squirting my eyes) see the ciliary localization, but isn't the majority of protein found in lower layers (maybe membranes again?) Please comment. If this is such a prominently expressing area, does the knockdown effect its morphology?

Dyrk1a is expressed generally near membranes, and we have now included this localization in the revised manuscript (Fig. 4A-B). We have also acknowledged this olfactory membrane expression in the revised manuscript (Line 540). We have observed some changes in the olfactory epithelium size, but have not rigorously quantified this and, as such, do not feel comfortable including this point in the manuscript.

The ciliary defect is striking, but should be quantified. Are cilia of ependymal cells nicely shown in figure 1J also effected by the knockdown? Or are any primary cilia in neuronal cells or elsewhere shortened? It's worth to know if there is a general or tissue specific ciliogenesis defect.

We now include a quantification of the ciliary defect severity in multiciliated cells by condition (Fig. 2D) and confirm there is a severe phenotype. We agree that an interesting future direction would be to assay the effect of *dyrk1a* loss on primary cilia in neurons (see also response to reviewer #1). We now comment that this will be important future work (Lines 188-190). We do not think that any potential effect on primary cilia is driving the brain size phenotype, however. A loss of primary cilia would result in a loss of hedgehog signaling (among other issues), and we would expect to see reduction in hedgehog target genes like *patched1* and *gli1* in the RNA-Seq data. We do not see these genes change. Further, a loss of hedgehog signaling would promote neural differentiation, and instead we see an increase in the relative proportion of neural progenitor cells compared to differentiated cells (Fig. 4F). Therefore, we think the cell cycle progression defect is the main driver of the brain size reduction and is most relevant to this manuscript.

The RNA-Seq read alignment show that CRISPR induced indels at the targeted locus, but is there any evidence for nonsense mediated decay of the dyrk1a mRNA? It does not seem to appear in the volcano plot. Is it possible that alternative initiation sites provide a read-through mechanism that doesn't much alter mRNA and protein levels? This may explain the relatively modest effect on

© 2020. Published by The Company of Biologists under the terms of the Creative Commons Attribution License (https://creativecommons.org/licenses/by/4.0/).

brain size as compared to the MO. Is there any evidence that spicing events in general are altered, as this is a proposed canonical function of Dyrk1a?

It is correct that we do not detect *dyrk1a* as a differentially expressed gene. When looking at the exon-level expression plot for *dyrk1a* (see below), we do not detect evidence for widespread nonsense mediated decay, nor compensatory exon usage. Therefore, we believe the phenotype is predominantly driven by frameshift inducing indels (i.e. mutations that result in loss of protein). Moreover, *DYRK1A* has been clearly demonstrated to be intolerant of genetic variation, and therefore, we expect most CRISPR-mediated indels to be loss of function. This conclusion is supported by loss of Dyrk1a antibody staining after CRISPR injection (Fig. S1). Rather, we think the MO- injected animals may have a stronger phenotype due to their disruption of both zygotic and maternally deposited *dyrk1a* mRNA, while CRISPR injections will only target zygotic expression. We now comment on this difference and the presence of maternal transcripts in the revised manuscript (Lines 116-119).

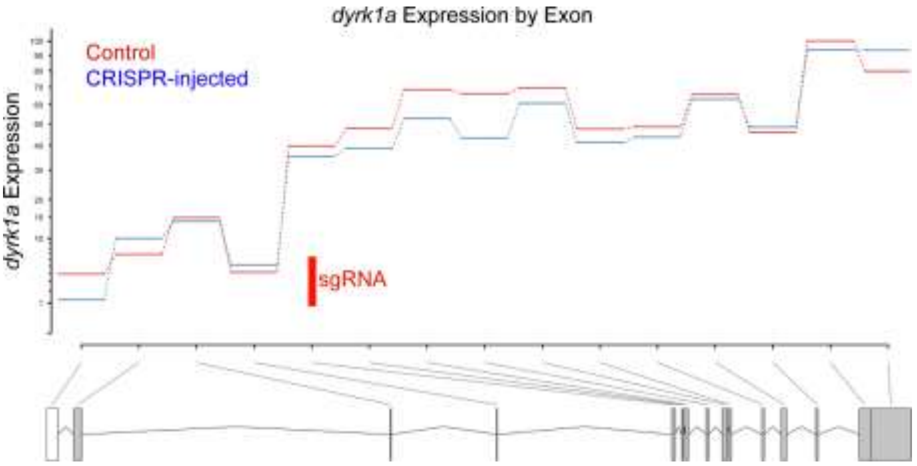

With regards to splicing, in the original manuscript we also analyzed the sequencing data for changes in exon-level expression and presented a supplementary table of these changes (Table S3). There are relatively few differences in exon usage, however, this analysis is quite underpowered as a much higher RNA-Seq coverage is required for assessing splicing difference accurately. We agree this is an important question for future work.

What is the proportion of the differentially regulated genes predominantly expressed in neuronal tissue? Are disease related genes among the regulated transcripts? It might be worth doing the GO analysis separately for up- or down-regulated transcripts.

We do not expect that the phenotypes caused by Dyrk1a loss of function will be restricted to brain tissue given the broad expression pattern of the transcript (Fig. 1). Therefore, we do not necessarily expect that changes will be brain-specific. Moreover, expression datasets for multiple tissues are not available at this stage of development, making this question difficult to answer. We are aware of the *Session et al.* dataset, but it is from adult *X. laevis* and so of unclear relevance to a developmental phenotype in *X. tropicalis*. Our RNA-Seq data was generated from brain tissue only, again making a comparison to other tissues difficult. We did, however, account for a neuronal expression background when conducting GO analyses to ensure that the input set of possible genes was not biasing the analyses.

We also assayed whether disease associated genes are among the differentially expressed genes. None of the current high-confidence ASD genes (*Satterstrom, et al., 2020*) are represented but three "Congenital anomalies of the kidney and urinary tract" genes (*Toka, et al., 2010*) are represented. These findings are of unclear significance and therefore we have not included them in the manuscript.

Given that genes can function in opposite directions (i.e. positive and negative regulation) it is not necessarily the case that genes functioning within the same pathway will be differentially expressed in the same direction. Therefore, it is not standard practice to do GO analyses

© 2020. Published by The Company of Biologists under the terms of the Creative Commons Attribution License (https://creativecommons.org/licenses/by/4.0/).

separately on the up- and down-regulated lists.
Consequently, we have retained the original integrated GO analysis. However, we would like to note that in general cell cycle genes, especially those marking S and M phase, showed increased expression (Fig. 3D), suggesting that either (1) the proportion of cells in these phases was increased or (2) that individual cells were upregulating these transcripts. Either way, this supports the idea that cells are stuck in cell cycle progression.

Fig 3E: What is the reason for leaving the previously validated genetic approach at this point and switch to pharmacological Dyrk1a inhibition? Is total brain size reduced in presence of the inhibitor as shown for the Mo/Crispr situation? Is PCNA staining increases on the Mo/Crispr injected side? Are there any know targets of Dyrk1a that may explain the cell cycle extension from a molecular perspective?

We now show that the pharmacological inhibitor also reduces brain size similarly to the genetic perturbations (Fig. 2G, I). We also now make it clear why we use this inhibitor to analyze cell cycle dynamics, namely so we can bypass early embryonic functions of Dyrk1a and temporally control loss of Dyrk1a to study its role in tadpole brain development (Lines 16-163). This is especially important because loss of Dyrk1a also induces cell death (Fig. 4J-L, Fig. S4A-B), so we wanted to temporally control inhibition so that we could see primary phenotypes in the brain instead of compensatory ones following prolonged cell death.

From a molecular perspective, we now show that Dyrk1a localizes to mitotic spindles (Fig. 4A-B). Ciliogenesis requires dynamic microtubule assembly and disassembly.
Mitosis also requires similar dynamic changes. Therefore, we hypothesize that loss of Dyrk1a affects mitotic spindle dynamics and stalls cells in M phase. Consistently, the mitotic cyclins B1 and B2 are up-regulated in the RNA-Seq data greater than 2 fold (Fig. 3D) and we see an increase in Phospho-Histone H3 staining, a marker for M phase (Fig. 4G-I). This would occur if cells were stuck in M phase, as a greater proportion of cells would express these genes. We now pose this microtubule hypothesis as a potential explanation linking both the ciliogenesis and brain size phenotypes (Lines 179-183).
Dyrk1a has previously been shown to affect microtubule dynamics by directly phosphorylating ß-tubulin in the context of neuronal dendrite development (Ori- McKenney et al., 2016), so we think this hypothesis is compelling.

Fig 4: This interesting data deserves rigorous quantification and statistics. Is PCNA staining affected (reduced) by overexpression of Dyrk1a? Does a kinase dead Dyrk1a-NES fail to rescue? From the methods section, it seems that plasmid DNA was injected, and I would expect a highly mosaic expression. The expression of the constructs should be monitored as the constructs are already GFP tagged.

We thank the reviewer for this important comment. We are now concerned about potential false negative results due to mosaic expression, like you point out. We did not assay GFP expression concurrently. Also, our antibody against endogenous Dyrk1a did not detect nuclear localized protein. Therefore, we have decided to remove these experiments from the manuscript. Instead, we now only show rescue with wildtype human protein not restricted to any particular cellular compartment (Fig. 2H-I).

Fig. S3: The nuclear size seems very different between A and C (scale bar similar) is this just by coincidence or a phenotype?

As per the above, we have decided to remove these experiments and instead focus the revised manuscript on the function of Dyrk1a in ciliogenesis and cell cycle control.

"Leverage this system to which Dyrk1a's function are relevant to ASD" – I see that this is a very nice model for structural brain anomalies, in particular microencephalopathy. But how could a psychiatric condition, such as ASD be modelled in tadpoles?

This point was alluding to a broader effort from our group (see *Willsey, et al., 2018 Cell*) to identify phenotypes in common from multiple ASD risk genes, as we think those phenotypes are more relevant to ASD pathobiology. Since this manuscript is only about 1 gene, we have

© 2020. Published by The Company of Biologists under the terms of the Creative Commons Attribution License (https://creativecommons.org/licenses/by/4.0/).

decided to remove this point from the text. With respect to modeling psychiatric conditions in tadpoles, we would like to emphasize that ASD and other psychiatric disorders, while diagnosed by higher order behavioral phenotypes, must have underlying biological changes, likely occurring during brain development (especially in neurodevelopmental disorders like ASD). Hence, the goal of our work in this model system of brain development is to identify these underlying biological changes and the corresponding molecular mechanisms, as that is a clear path to understanding the etiology of these conditions and to developing treatments.

Minor points:
The source of the Cas9-NLS protein should be given.

Thank you for catching this omission. The source (MacroLabs, UC Berkeley) and a relevant publication (Lingeman, Jeans, and Corn 2017) have been added (Lines 232, 252- 253).

Fig 2 F: ratios of injected vs. uninjected sides should be shown as log2-fold values. This will result in equidistant values < and > 1.

We appreciate this suggestion and have made this change to Fig. 2I.

Reviewer 3 Advance Summary and Potential Significance to Field:

This efficient study brings compelling evidence that dyrk1a is required for ciliogenesis, an important observation that was missed in human, mouse and Drosophila studies.

Reviewer 3 Comments for the Author:
This is a succinct yet convincing study on the role played by kinase dyrk1a in the context of Xenopus development. The authors intended, and succeed, to bring forth a novel pathomechanism for DYRK1A haploinsufficiency in humans which is associated with AUTOSOMAL DOMINANT (MIM614104) MENTAL RETARDATION 7 (MR7). The techniques employed to perform loss-of-function and rescue experiments in Xenopus tropicalis are adequate and the evidence brought forth about the localization of endogenous DYRK1A to cilia are solid.

Of course, it goes without mentioning but an acute depletion or permanent deletion of DYRK1A in a human cell line capable of ciliogenesis would be a welcome addition to validate the main findings from Xenopus embryos. this would significantly broaden the impact and reach of the conclusions that MR7 is a congenital syndromic ciliopathy. Besides this I have no major objections to its publication and suggest a couple of experiments that will strengthen the paper.

We thank the reviewer for their positive comments and suggestions. We would also like to note that in the revised manuscript we now highlight the potential role of Dyrk1a in mitotic spindle function, and that disruption of this function may result in the observed forebrain size phenotype. Therefore, we would like to be clear that we are not suggesting that DYRK1A-related conditions are exclusively driven by ciliopathy. Future research will validate key hypotheses in human neural progenitor cells but we believe that this work is beyond the scope of this manuscript.

1. What is the maternal contribution for DYRK1A? Can the other provide a developmental QPCR to document the onset of transcription.

There is evidence for maternal contribution of *dyrk1a* mRNA in *Xenopus* (see below graph of *dyrk1a* mRNA levels by developmental stage, from *Owens*, *et al*., accessed from Xenbase). We have now acknowledged this in the manuscript (Lines 116-119) and made the point that this could be why we observe stronger phenotypes with the morpholino, which will target maternal and zygotic expression, while CRISPR will only target zygotic.

© 2020. Published by The Company of Biologists under the terms of the Creative Commons Attribution License (https://creativecommons.org/licenses/by/4.0/).

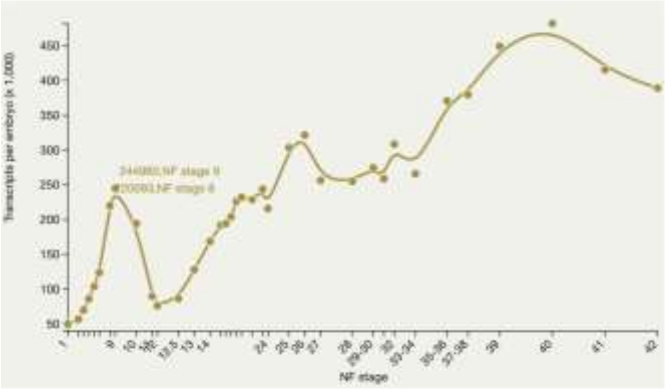

2. Being an established kinase, one would think that that a differential proteome performed on enriched phosphorylated proteins would have been a more accurate readout of the loss of DYRK1A. Instead authors chose to perform an RNAseq which is fine but is reflective of more indirect consequences. In this regard, the top 10 dysregulated genes (e.g. cfd, apoe, npas4) found by RNAseq must be validated by QPCR and /or WISH.

The main conclusion from the RNA-Seq data--namely that cell cycle progression may be inhibited--was derived from a systems analysis of multiple differentially expressed genes--and both the differentially expressed genes and the systems analysis were conducted with stringent thresholds and false discovery rates, and therefore, we believe it is unlikely that this is a false positive result, especially as our *in vivo* experiments clearly show defects in cellular proliferation.

3. What is the consequence of the loss of DYRK1A on motile beating cilia. Can their movement, or lack thereof, be documented. A simple dye assay should suffice.

We observe a dramatic loss of cilia and would assume this would lead to a motility defect. We think this experiment is not critical for the claims made in this manuscript, especially as we now highlight a potential role for Dyrk1a in mitosis, suggesting that our primary phenotype observed in the brain may not necessarily be due to lack of motile cilia.

Second decision letter

MS ID#: DEVELOP/2020/189290

MS TITLE: Neurodevelopmental disorder risk gene DYRK1A is required for ciliogenesis and brain size in Xenopus embryos

AUTHORS: Helen Rankin Willsey, Yuxiao Xu, Amanda Everitt, Jeanselle Dea, Cameron R.T. Exner, Arthur Jeremy Willsey, Matthew W. State, and Richard M. Harland
ARTICLE TYPE: Research Report

I am happy to tell you that your manuscript has been accepted for publication in Development, pending our standard ethics checks.

Reviewer 1

Advance summary and potential significance to field

All of my concerns for this manuscript were addressed and I fully support publication in Development.

© 2020. Published by The Company of Biologists under the terms of the Creative Commons Attribution License (https://creativecommons.org/licenses/by/4.0/).

Comments for the author

I recommend publication.

Reviewer 2

Advance summary and potential significance to field

The revised manuscript by Willsey et al. is now improved and more concise. My criticisms have been addressed, and much of the data that was criticized is now removed.
The additional data provided now focus on the cell-cyle associated functions of Dyrk1a and the main link to the ciliogenesis defect rests on the immunostaining showing a microtubular spindle staining.
Despite its slimmed down scope, I think the observations in the paper are still notable.

Comments for the author

The discussion now refers to the ciliogenesis defect as a potential molecular cause for the CAKUT phenotype in patients. I suggest to rephrase this paragraph. Most CAKUT cases are caused by mutations in transcription factors or GDNF signalling, while ciliogenesis defects can result in various forms of cystic kidneys, a distinct disease entity (See pubmed id 30172048 for a nice review on CAKUT).

Reviewer 3

Advance summary and potential significance to field

Same as previously with added insights into mitotic defects

Comments for the author

Why validating your RNAseq is too much to ask is beyond me.
I suppose those checks and balances don't apply here.

© 2020. Published by The Company of Biologists under the terms of the Creative Commons Attribution License (https://creativecommons.org/licenses/by/4.0/).
